# Novel PPAR Pan Agonist, ZBH Ameliorates Hyperlipidemia and Insulin Resistance in High Fat Diet Induced Hyperlipidemic Hamster

**DOI:** 10.1371/journal.pone.0096056

**Published:** 2014-04-23

**Authors:** Wei Chen, Shiyong Fan, Xinni Xie, Nina Xue, Xueyuan Jin, Lili Wang

**Affiliations:** 1 Beijing Institute of Pharmacology and Toxicology, Beijing, China; 2 International Center for Liver Disease Treatment, Beijing 302 Hospital, Beijing, China; Visva Bharati University, India

## Abstract

Effective and safe pharmacological interventions for hyperlipidemia remains badly needed. By incorporating the key pharmacophore of fibrates into the natural scaffold of resveratrol, a novel structural compound ZBH was constructed. In present study, we found ZBH reserved approximately one third of the sirtuin 1 (SIRT1) activation produced by resveratrol at in-vitro enzyme activity assay, directly bound to and activated all three peroxisome proliferator-activated receptor (PPAR) subtypes respectively in PPAR binding and transactivation assays. Moreover, ZBH (EC_50,_ 1.75 µM) activate PPARα 21 fold more efficiently than the well-known PPAR pan agonist bezafibrate (EC_50_, 37.37 µM) in the cellular transactivation assays. In the high fat diet induced hyperlipidemic hamsters, 5-week treatment with ZBH significantly lowered serum triglyceride, total cholesterol, LDL-C, FFA, hyperinsulinemia, and improved insulin sensitivity more potently than bezafibrate. Meanwhile, serum transaminases, creatine phosphokinase and CREA levels were found not altered by ZBH intervention. Mechanism study indicated ZBH promoted the expression of PPARα target genes and SIRT1 mRNA. Hepatic lipogenesis was markedly decreased via down-regulation of lipogenic genes, and fatty acid uptake and oxidation was simultaneously increased in the liver and skeletal muscle via up-regulation of lipolysis genes. Glucose uptake and utilization was also significantly promoted in skeletal muscle. These results suggested that ZBH significantly lowered hyperlipidemia and ameliorated insulin resistance more efficiently than bezafibrate in the hyperlipidemic hamsters primarily by activating of PPARα, and SIRT1 promotion and activation. ZBH thus presents a potential new agent to combat hyperlipidemia.

## Introduction

Widespread excessive diet and sedentary lifestyles have resulted in an exponential increase in hyperlipidemia worldwide [1], [2]. Additionally, many metabolic diseases, such as obesity and diabetes, are comorbidities associated with dyslipidemia [3]. Drugs that attenuate dyslipidemia are acutely important in the prevention of cardiovascular diseases [3], [4]. Within the past three decades, phenoxyalkylcarboxylic acid derivatives (fibrates) have been the most widely used drugs for hypertriglyceridemia [3], [5], [6]. By activating of peroxisome proliferator-activated receptor (PPAR) α, fibrates substantially reduce serum triglycerides and moderately elevate HDL-C levels, resulting in a small decrease in LDL-C levels [6], [7]. However the adverse effects of these drugs [hepatic toxicity (elevated serum transaminase), myopathy and cholelithiasis] limit their more widely using and, thus, discovery and characterization of novel scaffolds based small molecules are expected to avoid or reduce the adverse effects of current fibrate drugs and provide a superior profile compared with that of existing fibrates in dyslipidemia intervention.

**Figure 1:**
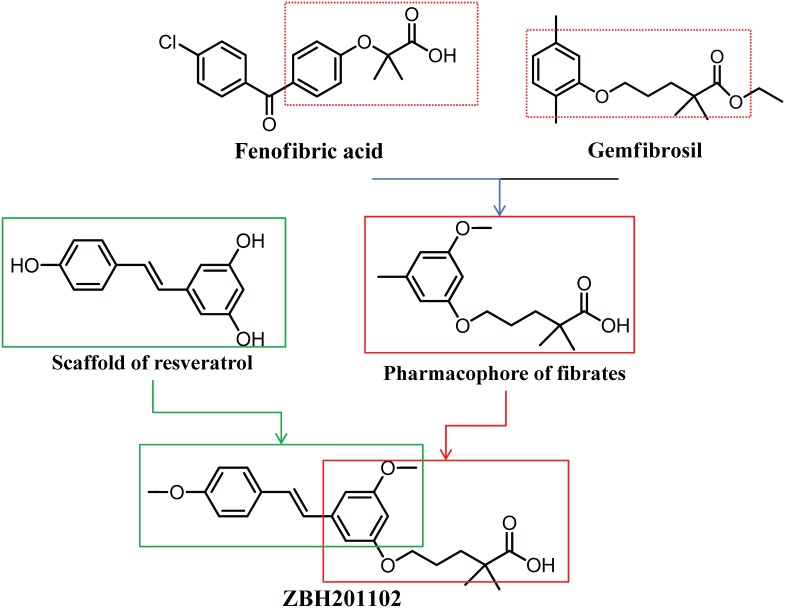
The structure of ZBH [(E)-5-(3-methoxy-5-(4-methoxystyryl) phenoxy)-2, 2- dimethylpentanoic acid] (MW, 406) and the philosophy of compound design. The key pharmacophore of fibrates and the scaffold of resveratrol were combined into ZBH.

Resveratrol (trans-3, 5, 4′-trihydroxystilbene) is a bioactive natural product found in grape, which exerts cardiovascular protective effects in age-associated chronic diseases through modulation of multiple targets [8], [9]. One target, sirtuin 1 (SIRT1), is an evolutionarily conserved NAD^+^ dependent protein deacetylase [8], [10]. SIRT1 activation promotes mitochondrial energy expenditure, prevented the onset of obesity, ameliorated dyslipidemia and reduced insulin resistance in rodents and humans [7], [11]. Furthermore, resveratrol also displays weak pan-activation to PPAR α, β and γ at in-vitro assays [12], [13]. The beneficial effects and safety profiles of resveratrol, as well its unique chemical structure makes resveratrol an ideal scaffold in synthesis of new molecules. To take advantage of the therapeutic potential of both fibrates and resveratrol, we sought to find structure diverse new anti-hyperlipidemic drugs with more effective anti-hyperlipidemic actions and less side effects, via incorporation of the fibrate carboxylic acidic head group which is responsible for hydrogen bonding interactions with the key amino acid residues in the ligand binding domain (LBD) of PPARα, with the scaffold of resveratrol that plays vital role in its beneficial effects (Figure 1). A series of α-alkyl-substituted aryloxyalkanoic acids had been designed, and synthesized in our institute previously [14]. The anti-hyperlipidemic action of compound ZBH is very impressive in dyslipidemic mice (Figure 1). Its oral LD_50_ is >1.2 g kg**^−^** ^1^ in Kunming mice; and this compound possesses favorable pharmacokinetics and excellent oral bioavailability (67%).

The hamster was recently proposed as a more ideal model than mice or rats for evaluating the efficacy of anti-hyperlipidemic agents as hamsters’ lipid metabolism and lipoprotein profile are relatively comparable to humans [15], [16]. Hamsters synthesize hepatic cholesterol and bile acids and also respond to dietary lipids like humans [15], [16], thus they are prone to hypercholesterolemia induced by excessive dietary cholesterol intake, while rats are resistant to this [15]. Moreover, hamsters also respond to treatment with a PPARα selective agonist fenofibrate in a manner similar to humans [15], [17], [18]. Therefore, to better forecast the anti-hyperlipidemic effect of ZBH in humans and disclose its mechanism of action, the impact of this compound was systematically evaluated on the high fat diet induced hyperlipidemic hamster. For comparison, bezafibrate was simultaneously chosen as a reference drug for its similar pan-activation to PPAR three isotypes.

## Materials and Methods

### Materials

ZBH (ZBH201102) and Bezafibrate were synthesized by the new drug design center of our institute. Purity and structure were confirmed by high performance liquid chromatography, mass spectrometry and ^1^H nuclear magnetic resonance (NMR). The structure of ZBH ((E)-5-(3-methoxy-5-(4-methoxystyryl)phenoxy) -2,2-dimethylpentanoic acid) is shown in Figure 1 and its synthesis has been previously described [14].

### Ligand Binding Assay with Surface Plasmon Resonance (SPR)

The binding affinities of ZBH for PPARα-LBD, PPARδ-LBD or PPARγ-LBD (Cayman chemical, MI, USA) were assayed using SPR-based Biacore T100 (GE Healthcare/Biacore, Uppsala, Sweden) as described previously [19], [20]. BIAevaluation software version 2.0.3 (GE Healthcare/Biacore) and steady state affinity fitting analysis were used to determine the equilibrium dissociation constant (KD) of compounds [20].

### In vitro Transactivation Assay

Transactivation of PPARs was analyzed in a cell-based luciferase reporter assay using HEK-293 cells transiently transfected to express hPPAR-GAL4 chimeric receptors with plasmids GAL4-hPPAR α, γ, or δ, and pUAS (5x)-tk-luc receptor vector as previously described [21]. 24 h after transfection, cells were treated with the indicated compounds, followed by the measurement of luciferase activity 24 h later. Mean values represent those obtained from at least three independent experiments performed in triplicate and normalized by the Renilla luciferase reading.

### SIRT1 Enzyme Activity Assay

SIRT1 enzyme activity was assayed with the SIRT1 Direct Fluorescent Screening Assay kit (Cayman chemical, MI, USA), according to the manufacturer’s instructions. Fluorescence was measured on an EnVision Multilabel Plate Reader (PerkinElmer, CA, USA). DMSO was used as a negative control and also as a solvent for resveratrol and ZBH.

### Animals and Treatment

Male golden Syrian hamsters (Mesocricetus auratus) weighing 90–110 g were purchased from Vital River Laboratory Animal Technology Co. Ltd. (Beijing, China). Animals were maintained on a 12-h day/night schedule with ad libitum access to nutrition and water. Animals were acclimated for 1 week before experimental animals were fed with a high fat diet (HFD) consisting of 10% coconut oil and 0.12% cholesterol. Blood samples were taken after overnight fasting from the suborbital sinus under light sevoflurane anesthesia on the fifth and tenth day for measurement of serum total cholesterol (TC) and triglyceride (TG). Hamsters in which serum lipids were significantly increased were selected and randomly divided into 6 groups (n = 11–12 each) according to their initial body weight and baseline serum TC and TG levels, and received bezafibrate (25 or 50 mg kg**^−^** ^1^ per day), ZBH (6.25, 12.5 or 25 mg kg**^−^** ^1^ per day), or vehicle (model control, MC). The dose selection of ZBH was based on previous pilot study. Hamsters with standard rodent diet were used as normal control. Compounds were dissolved in DMSO and suspended in 0.5% methylcellulose and administered by gavage once a day for 5 weeks. Individual body weight and cage food consumption were measured every two days. Dynamic serum lipid monitoring was performed weekly and blood samples were taken from the retro-orbital venous plexus under light ether anesthesia using a glass capillary tube after overnight fasting. Then the animal were sacrificed by cervical decapitation under anesthesia, and the liver, heart, epididymal white adipose tissue (eWAT) and gastrocnemius muscle were excised, rapidly frozen in liquid nitrogen and maintained at −80°C until analysis.

Ethic statement: All animal handling and experiments were performed strictly in accordance with the recommendations in the Guide for the Care and Use of Laboratory Animals of the National Institutes of Health. The protocol was approved by the Animal Experimental Ethics Committee of Beijing Institute of Pharmacology and Toxicology.

### Metabolic Studies

Serum TC, TG and free fatty acids (FFA) were measured by using commercial kit (Nanjing Jiancheng, Bioengineering Institute, Nanjing, China) as previously described [21]. Serum glucose, low-density lipoprotein cholesterol (LDLc) and high-density lipoprotein cholesterol (HDLc) were measured with the Hitachi 917 automated biochemistry analyzer (Roche diagnostics, Indianapolis, USA). Serum insulin was quantified by ELISA using a hamster insulin assay kit (Crystal Chem Inc., IL, and USA). The insulin sensitivity index (ISI) was calculated by the values of fasting blood glucose (FBG) and fasting blood insulin (FBI). ISI = 1/(FBG×FBI) 1000 [21].

### Determination of Hepatic Lipids

For determination of TG and TC in liver, 100 mg frozen tissue was homogenized with TissueLyserII (Qiagen, Germantown, MD, USA) in 2 ml chloroform/methanol (2∶1, v/v) for 16 h, after which 2% KH_2_PO4 was added and the solution was centrifuged. After evaporation of the chloroform under nitrogen, lipid samples were resuspended in isopropyl alcohol, and TG and TC content was determined by enzymatic assay [21].

### Histopathological Examination

Liver samples were resected and fixed with 10% formaldehyde phosphate buffer saline (PBS, pH = 7.4), and embedded in paraffin, sectioned, stained with hematoxylin/eosin (HE) and analyzed by microscopy and morphometry [21]. Frozen liver samples were used to perform hepatic lipid specific staining with Oil Red O staining.

### Quantitative Real-time PCR

To determine the relative mRNA expression levels of lipid metabolism-related genes, total RNA was isolated from the liver, white adipose tissue (WAT) or muscle and real-time PCR was performed with the ABI PRISM 7300 sequence detection system (Applied Biosystems, Warrington, UK) as previously described [21]. The relative amount of all mRNAs was calculated using the comparative C_T_ method. Target gene expression is presented relative to β-actin expression.

### Statistical Analysis

All results are expressed as mean±SE. For multiple comparisons statistical analysis was performed either by one-way ANOVA followed by Tukey multiple comparison tests or by 2-way ANOVA plus Repeated Measurements with SPSS 13.0 software. P<0.05 was considered to be statistically significant.

## Results

### ZBH Directly Interacts with PPARs–LBD

**Figure 2:**
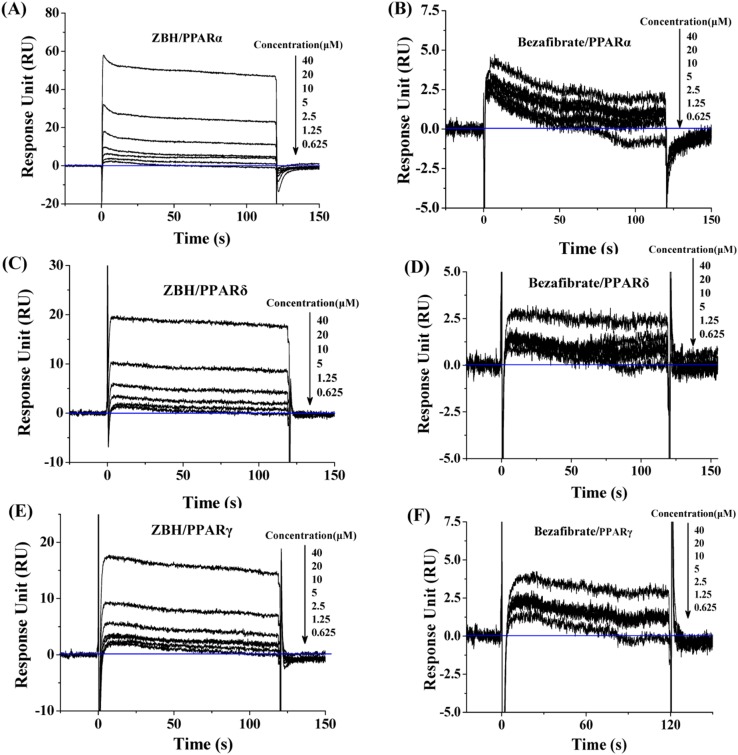
Kinetic analyses of ZBH (A, C, E) and bezafibrate (B, D, F) binding to PPARα (A, B), hPPARδ (C, D) and hPPARγ (E, F) measured by SPR (Biacore T100). Representative sensorgrams obtained from injections of ZBH and bezafibrate at different concentrations. The ligands were injected for 120-LBDs on the CM5 sensor chip.

ZBH association with PPARs-LBD was evaluated by Biacore SPR experiments. We found that the response units (RU) increased with increasing concentrations of ZBH and bezafibrate, indicating that both bound directly, in a dose-dependent manner, to PPARα, PPARδ and PPARγ-LBD (Figure 2). The dissociation constants (KD) suggest similarly direct and robust binding of both compounds to the three PPAR subtypes (Table 1). However, the RUs of ZBH are higher than those of bezafibrate for all subtypes, demonstrating that ZBH is a non-selective ligand of all three PPAR subtypes with higher affinity for PPARα-LBD than bezafibrate.

**Table 1:** Kinetic parameters for the binding of ZBH to PPARs-LBD.

	K_D_ (M)		
	hPPARα-LBD	hPPARδ-LBD	hPPARγ-LBD
**Bezafibrate**	**9.29×10^−7^**	**2.38×10^−6^**	**4.46×10^−6^**
**ZBH**	**8.26×10^−7^**	**4.17×10^−6^**	**3.90×10^−6^**

### The PPARs Transcription Agonism of ZBH

**Figure 3:**
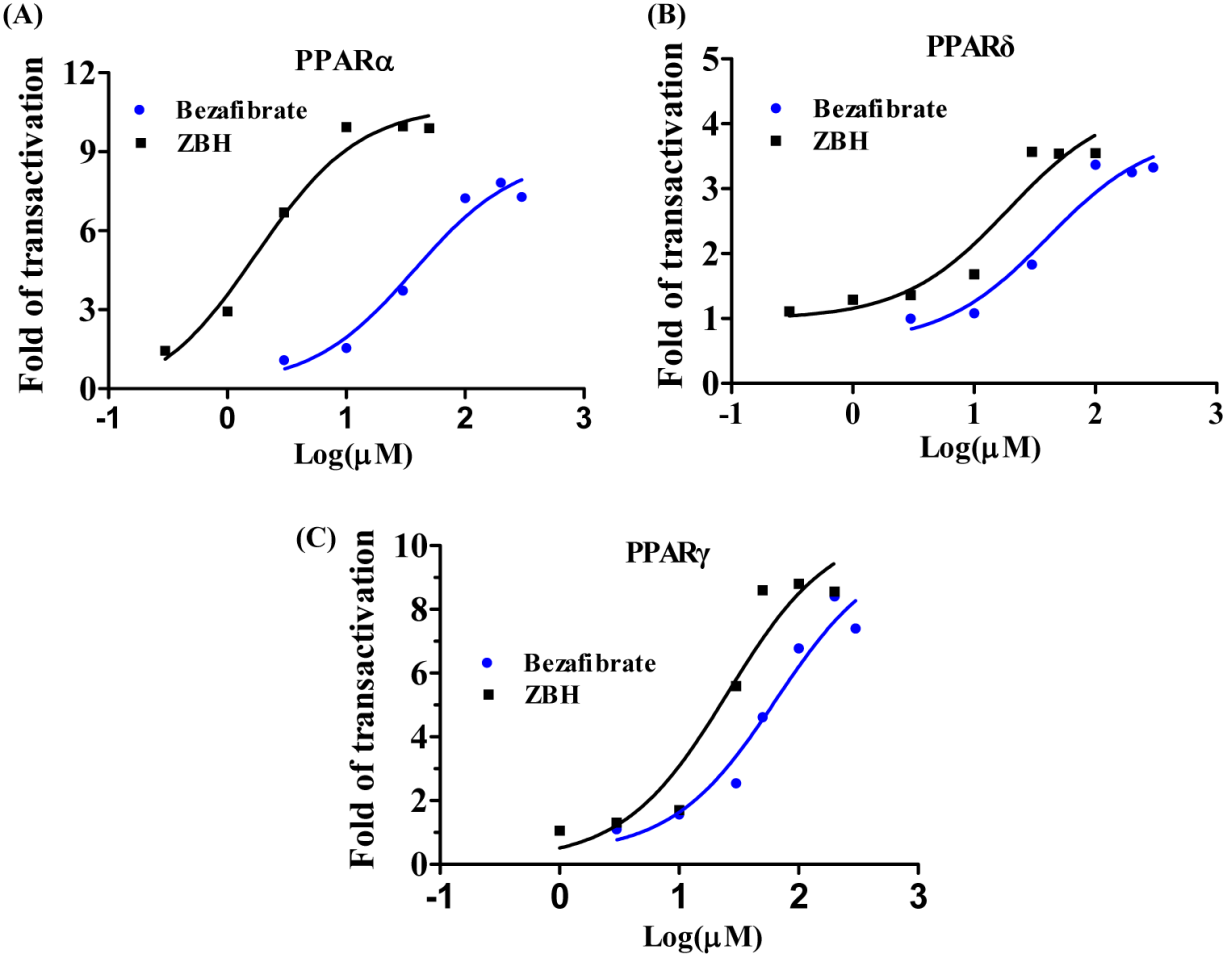
ZBH activation of hPPARα (A), hPPARδ (B) and hPPARγ (C). HEK-293 cells transiently transfected to express hPPAR-GAL4 chimeric receptors with plasmids GAL4-hPPAR α, γ, or δ, and pUAS (5x)-tk-luc receptor vector. 24 h after transfection, cells were treated with the indicated compounds at various concentrations followed by the measurement of luciferase activity 24 h after treatment. The results shown are the mean values obtained from at least three independent experiments performed in triplicate normalized by the Renilla luciferase reading.

As previously reported [22], bezafibrate achieved weak and dose-dependent pan-agonism of the three PPAR subtypes at comparable doses (37.37 µM, 40.30 µM and 64.76 µM for PPARα, PPARδ and PPARγ respectively) (Figure 3 and Table 2). ZBH achieved approximately 11-fold maximal activation of PPARα and an EC_50_ (1.75 µM) 21 fold greater than that of bezafibrate. Like bezafibrate, ZBH also exhibits weak and dose-dependent activation to PPARδ and PPARγ at higher concentrations; however its PPARα activation exceeds PPARδ and PPARγ activation by 11 and 14 fold respectively (Figure 3 and Table 2).

**Table 2:** In vitro transactivation activity of ZBH in different hPPAR subtypes.

Compound	EC_50_ (µM)		
	hPPARα	hPPARδ	hPPARγ
**Bezafibrate**	**37.37**	**40.30**	**64.76**
**ZBH**	**1.75**	**19.19**	**25.35**

Mean values represent those obtained from at least three independent experiments performed in triplicate and normalized to Renilla luciferase reading.

### Activation of SIRT1 by ZBH

**Figure 4:**
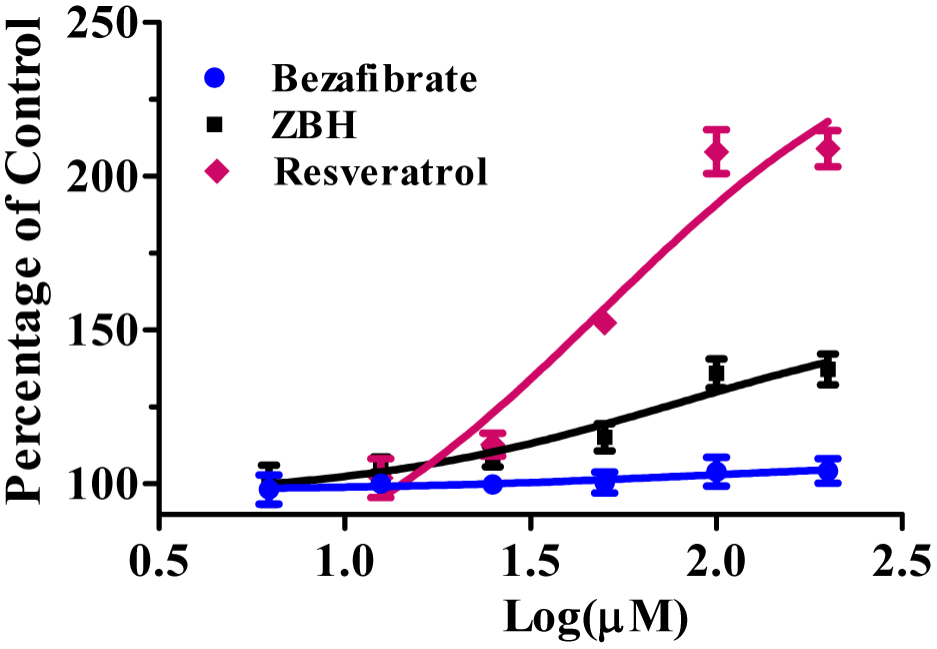
Activation of SIRT1 by ZBH. SIRT1 enzyme activity was measured after treatment with ZBH and Resveratrol, and the fluorescence was tested on EnVision Multilabel Plate Reader. Values are mean±SE, n = 3.

An enzyme bioactivity assay confirmed that ZBH could activate SIRT1. The maximal activation was roughly one third of that achieved by resveratrol (135.94% vs. 207.98%), whereas the EC_1.5_ of ZBH (EC_1.5_ = 78.6 µM) is close to that of resveratrol (EC_1.5_ = 48.9 µM) (Figure 4). We found the EC_1.5_ and maximum activation of resveratrol to be equivalent to previously reported data (46.2 mM and 201% respectively) [23].

### Impact of ZBH on Body Weight Gain and Physical Parameters in Hyperlipidemic Hamsters

**Figure 5:**
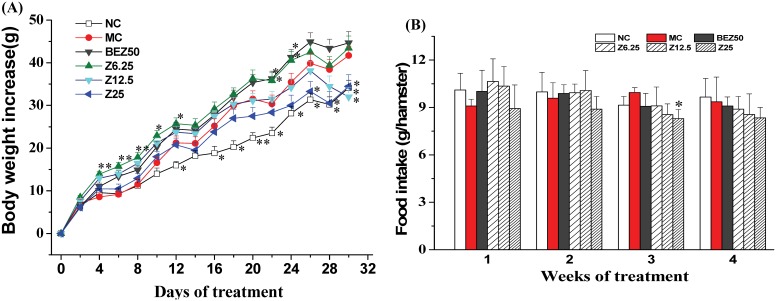
Effect of ZBH on body weight (A) and food intake (B) in hyperlipidemic hamsters, which was calculated for individual hamster and then averaged. Hyperlipidemic hamsters were given indicated dose of bezafibrate (BEZ) or ZBH (Z) for 5 weeks. Values are mean±SE; n = 8 for NC, n = 11–12 for other groups. *P<0.05, **P<0.01 vs. MC group. NC, normal control; MC, model control; BEZ50, Bezafibrate 50 mg kg**^−^** ^1^; Z6.25, ZBH 6.25 mg kg**^−^** ^1^; Z12.5, ZBH 12.5 mg kg**^−^** ^1^; Z25, ZBH 25 mg kg**^−^** ^1^.

As depicted in Figure 5A, the body weight of hamsters in model control (MC) group had significantly increased within 2-weeks, in comparison to hamsters fed a normal diet (NC group). This weight gain was maintained till the end of the experiment and was not altered by bezafibrate treatment. However, hamsters administered high dose (25 mg/kg) ZBH gained less weight from the second week of treatment and throughout the two weeks follow up (Figure 5A).

Impact of ZBH on food intake is negligible; although it showed a relative decreasing tendency in a dose-dependent manner at the last 2 weeks of treatment, but statistical difference is not present (Figure 5B). The brown and white adipose tissue located in subcutaneous, retroperitoneal, and mesenteric compartments was also decreased considerably by high dose ZBH (25 mg kg**^−^** ^1^) (Table 3). In addition, the weight of the liver of hamsters treated with ZBH also showed increasing tendency (7%∼17%) after 5 weeks. PPARα agonists often induce increases in the size of the liver and kidney [24]–[26]; however ZBH treatment appears to only slightly impact the size of the liver (Table 3). Meanwhile, serum aspartate aminotransferase (GOT), alanine aminotransferase (GPT), creatine phosphokinase (CK) and creatinine levels were found not altered by ZBH intervention in current study (Figure S1).

**Table 3:** Effects of ZBH on physical parameters in hyperlipidemic hamsters.

	NC	MC	Bezafibrate	ZBH		
			50 mg/kg	6.25 mg/kg	12.5 mg/kg	25 mg/kg
Subcutaneous WAT(g)	5.44±0.27*	7.42±0.39	7.48±0.47	7.28±0.32	6.53±0.42	5.19±0.12**
Retroperitoneal WAT(g)	9.94±0.58**	14.14±0.69	13.93±0.44	13.93±0.82	12.63±0.66	10.43±0.39**
iBAT(g)	0.22±0.01	0.25±0.02	0.24±0.02	0.23±0.01	0.21±0.01	0.19±0.01
Liver(g)	4.88±0.17**	7.25±0.28	7.04±0.21	7.78±0.23	7.77±0.31	8.52±0.45
Kidney(g)	0.96±0.04	0.99±0.10	1.02±0.11	1.01±0.08	0.95±0.09	0.94±0.05

Compounds or vehicle were administered for 5 weeks at the indicated doses. NC, normal control; MC, model control; WAT, white adipose tissue; iBAT, interscapular brown adipose tissue. Values are mean±SE. n = 8 for NC, n = 11–12 for other groups.

*P<0.05, **P<0.01, vs. MC group.

### Lipid Lowering Effect of ZBH in Hyperlipidemic Hamsters

**Figure 6:**
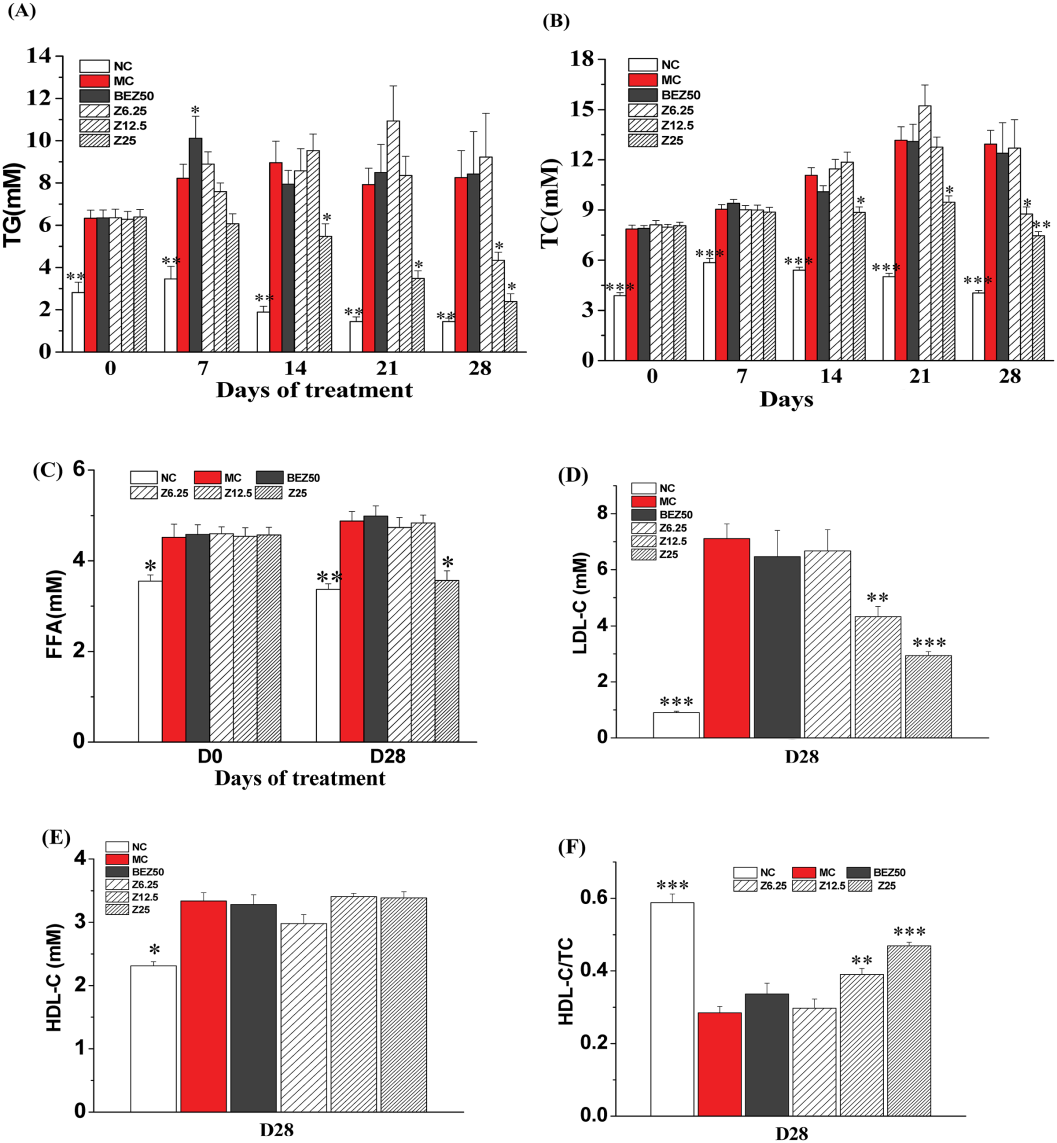
Effect of ZBH on serum lipids in hyperlipidemic hamsters. Serum TG (A), TC (B), FFA (C), LDL-C (D), HDL-C (E) were measured in hyperlipemic hamsters following 10 days of HFD and then orally gavage with the indicated dose of bezafibrate (BEZ) or ZBH (Z) for 5 weeks, and HDL-C/TC(F) was calculated accordingly. Values are mean±SE; n = 8 for NC, n = 11–12 for other groups. *P<0.05, **P<0.01, **P<0.001 vs. MC group. NC, normal control; MC, model control; BEZ50, Bezafibrate 50 mg kg**^−^** ^1^; Z6.25, ZBH 6.25 mg kg**^−^** ^1^; Z12.5, ZBH 12.5 mg kg**^−^** ^1^; Z25, ZBH 25 mg kg**^−^** ^1^.

We observed 10-days of HFD dramatically increased serum TG (2.3 fold), TC (2.1 fold) and FFA (1.4 fold) in hamsters (Figure 6A–C), and metabolic dyslipidemia exacerbated with time. Serum LDLc had increased nearly 8 fold over normal control levels by the end of the experiment, accounting for the strikingly increased serum TC (Figure 6B and D). The HDLc/TC ratio was thus reduced by almost half (0.59±0.02 vs. 0.28±0.02, P<0.001) (Figure 6F). Severe obesity and insulin resistance also emerged within 7 weeks (Figure 5A and 8). The increase in circulating triglycerides and induced hyperlipidemia within the HFD hamster model corresponded with the pattern of hyperlipidemia progression observed in humans with elevated TG and a reduced HDLc/LDLc ratio [16]. Administration of 12.5 or 25 mg kg**^−^** ^1^ ZBH induced a time and dose-dependent decrease in serum TG, TC and LDLc, and a dose-dependent increase in HDLc/TC (Figure 6). Serum FFA also lowered remarkably after one-week treatment with 25 mg kg**^−^** ^1^ ZBH and it lasted until the end of the experiment (Figure 6C). Five weeks of administration of 50 mg kg**^−^** ^1^ bezafibrate, higher than the clinically relevant dose (10 mg kg**^−^** ^1^) [27], did not alter these serum lipid parameters in hyperlipidemic hamsters under same conditions (Figure 6).

**Figure 7:**
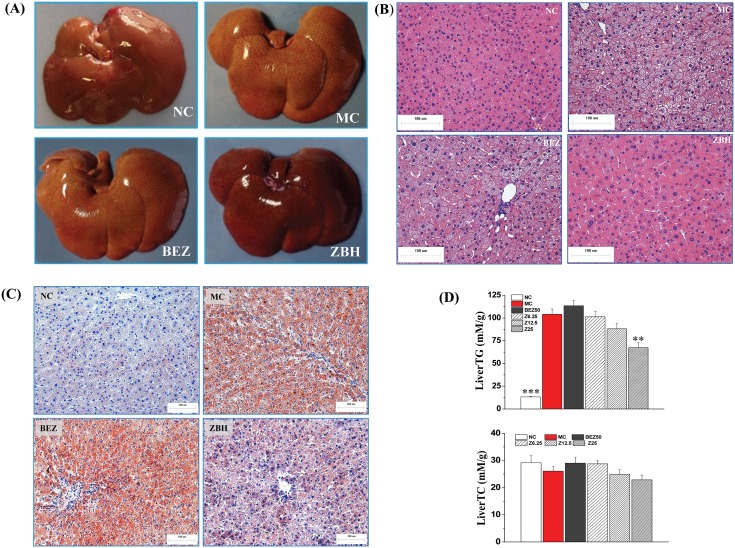
Effects of ZBH on hepatic lipids accumulation in hyperlipidemic hamsters. (**A**) Photograph of liver. The liver of MC hamster is liparoid yellow and swelling is apparent when compared to a normal hamster (NC). ZBH treatment reduces these effects more substantially than BEZ. (**B**) **and** (**C**) Photomicrograph of histological examination based on hematoxylin-eosin staining and Oil Red O staining respectively. Hepatic steatosis is evident in the MC group (Original magnification: 200×). (**C**) Hepatic TG and TC contents. Values are mean±SE; n = 8 for NC, n = 11–12 for other groups. *P<0.05, **P<0.01, ***P<0.001 vs. MC group. NC, normal control; MC, model control; BEZ50, Bezafibrate 50 mg kg**^−^** ^1^; Z6.25, ZBH 6.25 mg kg**^−^** ^1^; Z12.5, ZBH 12.5 mg kg**^−^** ^1^; Z25, ZBH 25 mg kg**^−^** ^1^.

**Figure 8:**
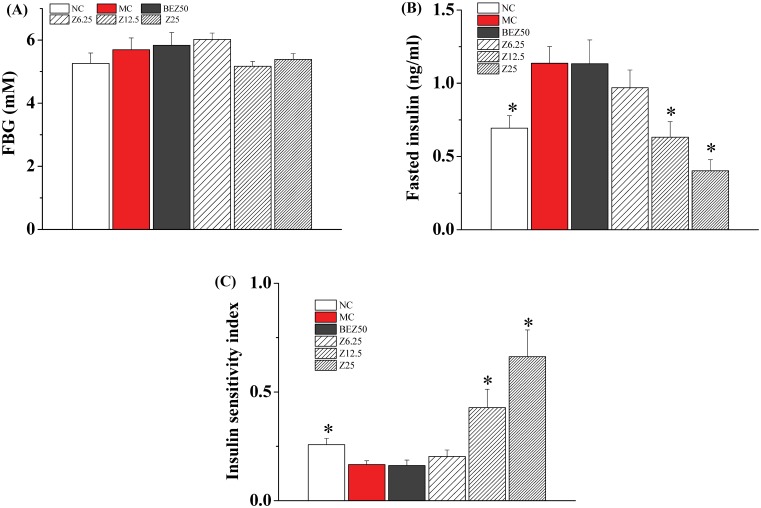
Effect of ZBH on serum glucose (A), serum insulin (B) and ISI (C) in hyperlipidemic hamsters. At the end of the 5-week experiment, hyperlipidemic hamsters were bled retroorbitally and serum glucose and insulin was measured. Values are mean±SE; n = 8 for NC, n = 11–12 for other groups. *P<0.05, **P<0.01 vs. MC group. NC, normal control; MC, model control; BEZ50, Bezafibrate 50 mg kg**^−^** ^1^; Z6.25, ZBH 6.25 mg kg**^−^** ^1^; Z12.5, ZBH 12.5 mg kg**^−^** ^1^; Z25, ZBH 25 mg kg**^−^** ^1^.

### Anti-hepatic Steatosis Effect of ZBH in Hyperlipidemic Hamsters

The liver of MC hamsters appeared liparoid yellow, swollen, and had lost its normal kermesinus and luster (Figure 7A), histopathological examination revealed hypertrophy of hepatocytes and hepatic steatosis (Figure 7 B and C). In accordance with this, hepatic TG contents of hamsters on a high fat diet reached 9 fold that of normal hamsters (P<0.001, Figure 7D), further confirmed the existence of hepatic steatosis. Unlike bezafibrate treatment, which did not ameliorate hepatic steatosis, ZBH greatly lowered hepatic TG contents in a dose-dependent manner, and significantly lowered hepatic lipid droplet accumulation, returning the normal hepatic morphology (Figure 7). These results strongly suggest that ZBH reduces fat levels in liver.

### Insulin Sensitizing Effect of ZBH in Hyperlipidemic Hamsters

HFD feeding significantly increased serum insulin levels, possibly as a result of elevated serum lipids and induced mild peripheral insulin resistance, as reflected by markedly elevated fasting serum insulin and reduced ISI (Figure 8). ZBH treatment led to a dramatic, dose-related decrease in insulin levels with a maximum decrease of 84% at 25 mg kg**^−^** ^1^, and resulted in a significant improvement in insulin sensitivity (Figure 8A and C). Fasting blood glucose levels in 12.5 and 25 mg kg**^−^** ^1^ ZBH treated hamsters was comparative to that of NC hamsters (Figure 8B). However, bezafibrate treatment did not impact serum insulin and ISI.

### Effect of ZBH on Gene Expression

**Figure 9:**
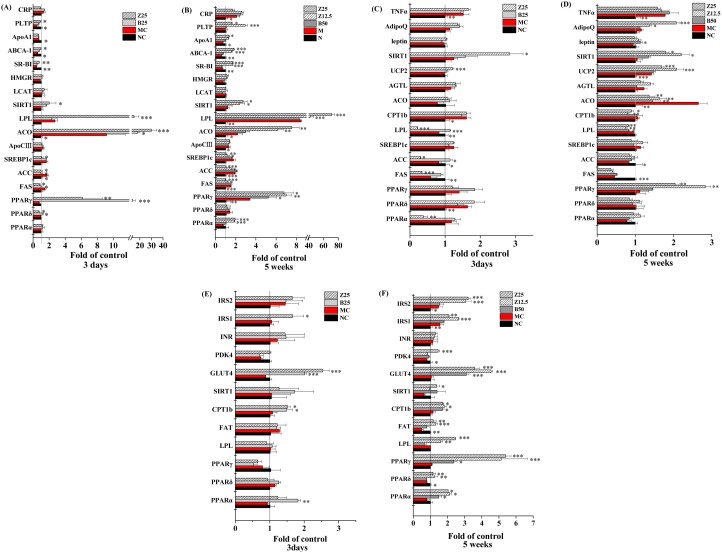
Effects of ZBH on gene expression in hyperlipidemic hamsters. Relative gene expression of the indicated genes in the liver (A and B), WAT (C and D) and skeletal muscle (E and F) of hyperlipidemic hamsters following 3 days (A, C and E) and/or 5 weeks (B, D and F) of ZBH or BEZ administration was determined by QPCR. Results are normalized to β-actin and expressed as mean fold increase of mRNA ±SD compared to the normal hamsters (NC). n = 3. *P<0.05, **P<0.01, ***P<0.001 vs. MC group. NC, normal control; MC, model control; B25, Bezafibrate 25 mg kg**^−^** ^1^; B50, Bezafibrate 50 mg kg**^−^** ^1^; Z12.5, ZBH 12.5 mg kg**^−^** ^1^; Z25, ZBH 25 mg kg**^−^** ^1^.

To elucidate the mechanism by which ZBH improves metabolic dysfunction in the hyperlipidemic hamster, gene expression profiles of the liver, adipose and skeletal muscle were analyzed in hamsters administered ZBH for a short term (3 days) or chronically (5 weeks). Short-term administration of 25 mg kg**^−^** ^1^ ZBH significantly increased expression of key enzymes involved in TG catabolism (lipoprotein lipase (LPL)), fatty acid oxidation (acyl-CoA oxidase (ACO)) and PPARγ in the liver, and significantly decreased expression of genes mediating endogenous fatty acid synthesis (sterol response element binding protein 1c (SREBP1c), fatty acid synthase (FAS) and acetyl CoA carboxylase (ACC)) (Figure 9A). Meanwhile, upregulation of ATP-binding cassette transporter A 1 (ABCA-1), scavenger receptor class B type I (SR-BI) and phospholipid transfer protein (PLTP) expression suggests that reverse cholesterol transport to the liver was increased. Short-term bezafibrate (25 mg/kg) treatment did not affect the expression of all genes altered by ZBH, but did impact the expression of ACO, PPARγ, FAS and ACC.

The gene expression profile in adipose tissue indicated that representative PPARγ target genes such as leptin, AdipoQ (adiponectin) and TNFα were not altered by short-term ZBH treatment, but the mRNA level of UCP2 (involved in energy uncoupling) was significantly increased, whereas those genes implicated in fatty acid uptake and synthesis such as LPL, FAS, ACC and PPARα were down-regulated by ZBH (Figure 9C). Short-term Bezafibrate treatment did not impact the expression of these genes in adipose tissue. In skeletal muscle, key genes for fatty acid oxidation (carnitine palmitoyl transferase I b (CPT1b)) and glucose uptake (glucose transporter 4 (GLUT4)) were similarly up-regulated by short-term ZBH or bezafibrate administration; and insulin receptor substrate (IRS) 1 mRNA expression was up-regulated only by ZBH (Figure 9E).

The expression profile after chronic ZBH and bezafibrate treatment is similar to that resulting from short-term administration, but reflects more potent alterations in gene expression. In the liver ABCA-1 and PPARα expression increased (Figure 9B). In adipose tissue, chronic ZBH treatment no longer affected PPARα expression or fatty acid synthesis (FAS and ACC), but inhibited fatty acid oxidation (ACO and CPT1b), increasing expression of AdipoQ and up-regulating UCP2 and PPARγ expression (Figure 9D). In skeletal muscle, in addition to CPT1b, GLUT4 and IRS1, the expression of LPL, fatty acid translocase (FAT), pyruvate dehydrogenase kinase isoform 4 (PDK4), IRS2 and all three PPAR subtypes were all markedly upregulated with extended administration of ZBH, particularly PPARγ (Figure 9F). Interestingly, SIRT1 mRNA expression was upregulated not only in the liver, but also in adipose tissue and skeletal muscle after both short-term and chronic ZBH treatment (Figure 9A–F), but bezafibrate treatment did not up-regulate SIRT1, consistent with the results of the in vitro SIRT1 enzyme assay, in which bezafibrate also did not impact SIRT1.

## Discussion

Not completely similar to bezafibrate and resveratrol, they both activate PPAR three subtypes weakly (Figure S2) [22], ZBH was categorized as a PPAR pan-agonist with 11 fold greater affinity for PPARα than the other two PPAR subtypes. Moreover, ZBH activates PPARα 21 fold more potently than bezafibrate, and its powerful PPARα activation was further manifested on regulating expression of PPARα target genes in vivo. Molecular docking studies revealed that ZBH could interact with the key amino acid residues (Ser280, Tyr464, Tyr314) in the LBD essential for activation of PPARα and may bind to the receptor in a different mode from that of fenofibric acid (the active form of fenofibrate) and bezafibrate [28]. The two phenyl rings of fenofibric acid were docked into the hydrophobic pocket (also known as “benzophenone” pocket) formed by the helices 3, 6, and 10 adjacent to the AF-2 helix. Whereas the two phenyl rings of ZBH were docked into the hydrophobic pocket formed by the helices 2′, 3, and β sheet, the gem-dimethyl substituents were directed into a lipophilic pocket bounded by Phe273, Gln277, Val444, and Leu456, a region at the top end of the “benzophenone” pocket.

ZBH potently reduced the metabolic dysfunctions in the hyperlipidemic hamsters after only 1 week of administration. Dramatic increases in serum TG, TC and FFA, caused by the HFD, were reduced by administration of ZBH. LDLc and the HDLc/TC ratio was decreased and increased respectively within five weeks of administration. Hepatic lipids were simultaneously reduced and liver steatosis was effectively ameliorated. Adipose tissue content was also reduced. An increase in the relative liver weight, evidence of activation of PPARα in rodents [25], appeared in hamsters given the highest dose of ZBH (17% increasing relative to that of MC), but was absent in bezafibrate treated hamsters. This species-specific response may be related to proliferation of peroxisomes via induction of a set of genes encoding peroxisomal fatty acid oxidation and biogenesis [24], [29]. To some extent, this also further signifies that PPARα was not effectively activated by current dose bezafibrate. Thus consistent with in-vitro PPARα transactivation data, the in-vivo results in hamster further corroborated the powerful potency of ZBH relative to the clinical widely used drug bezafibrate in ameliorating dyslipidemia.

In accordance with the powerful pharmacodynamics of ZBH, in-vivo gene expression analysis corroborated that ZBH achieved strong agonism of PPARα rather than PPARδ and PPARγ. Both short-term and chronic treatment with ZBH regulated PPARα target gene expression in the liver of the hyperlipidemic hamsters. PPARα, as a ligand dependent transactivator, directly controls expression of a comprehensive set of genes that regulate lipid catabolism, and lipoprotein synthesis and metabolism. By elevating LPL, triglyceride-VLDL lipolysis was increased and VLDL clearance was improved. Thereby atherogenic LDLc, and serum and hepatic TG were reduced. Meanwhile, the reduction in TG is attributed to enhancement of fatty acid β-oxidation via significantly up-regulated ACO, and reduced endogenous fatty acid synthesis by down-regulating SREBP1c, FAS and ACC. Energy uncoupling was simultaneously induced in adipose tissue. This reduction in the amount of fatty acids available for TG synthesis and storage in white adipose tissue is consistent with the significantly decreased adipose pad and hepatic steatosis observed in ZBH treated hamsters. Reduction in serum cholesterol may occur primarily through increased reverse cholesterol transport by accelerating the efflux of cholesterol from peripheral tissues, and promoting its uptake into the liver through a pathway involving increased expression of ABCA-1, SR-BI and PLTP; where cholesterol is excreted in the bile as free cholesterol or as bile salts. ZBH induced a dramatic decrease in serum LDL-C, and raised the HDL-C/TC ratio in the hyperlipidemic hamsters, similar to the human response to human lipoprotein metabolism.

We also found that the marked induction of hepatic PPARα gene seems to be mediated indirectly and secondarily to responses to the metabolic ameliorations, as it was observed only after chronic treatment. Meanwhile we found that fatty acid synthesis was inhibited at the initial stage in adipose tissue when hyperlipidemia was present, whereas this inhibitory effect disappeared when serum lipids were recovered after 5-week ZBH treatment. The elevated expression of PPARγ and adiponectin (the beneficial adipokine) in chronic ZBH treatment also may be a collateral effect. In skeletal muscle, although only fatty acid oxidation (CPT1b) was enhanced initially, fatty acid uptake (LPL, FAT) and oxidation were all increased after chronic ZBH administration; as confirmed by increasing in PDK4 expression. Thus, gene expression in hamsters consistently suggested that ZBH markedly decreased endogenous fatty acid synthesis in the liver and increased fatty acid uptake and oxidation in the liver and skeletal muscle.

Previous studies had demonstrated that SIRT1 activation could enhance the ability of organisms to consume fat and use mitochondrial respiration to optimize energy harvesting [10], [30], [31], and resveratrol could stimulate the expression of SIRT1 mRNA in adipocytes and increase SIRT1 activity in rodents and humans [32]. Inclusion of the scaffold of resveratrol in the structure of ZBH engendered the compound with approximately one third of the SIRT1 activation produced by resveratrol when assayed at the in-vitro level. In the hamster, ZBH induced significant increases in the SIRT1 mRNA expression within the main insulin target tissues. SIRT1 mediated deacetylation activates PGC-1α [33], and may thus facilitate ZBH’s activation of PPARα and full transcriptional induction of PPARα target gene expression. Hepatic deletion of SIRT1 impairs PPARα activity, decreases fatty acids oxidation, and results in hepatic steatosis and inflammation in response to high-fat feeding [34]. In contrast, even moderate SIRT1 overexpression in mice could protect against the development of hyperglycemia, fatty liver, and metabolic diseases [35], [36]. Moreover, studies also had demonstrated that the mRNA and protein expression of SIRT1 is reduced in obese mice and humans with dyslipidemia. The present results thus suggesting that ZBH mediated amelioration of dyslipidemia is at least partially dependent upon the up-regulation of SIRT1 expression [37].

Besides hyperlipidemia, this hamster model also developed mild insulin resistance, particularly in the liver, manifested by elevated basal insulin excursion and lowered ISI. This observation is in line with report of a strong link between the development of dyslipidemia and diabetes. ZBH, but not bezafibrate, prevented mild hyperinsulinemia and reduced insulin insensitivity in the high fat-fed hamsters. Which may mainly result from PPAR activation, GLUT4 and IRS mRNA in skeletal muscle were rapidly up-regulated within 3 days, and this up-regulation became more prominent as chronic treatment induced PPARγ expression in insulin sensitive target tissues, particularly skeletal muscle. Significantly increased expression of adiponectin in hamster adipose tissue may also partially contribute to this. We also found that 10–100 µM ZBH could dramatically up-regulate adiponectin expression in 3T3-L1 adipocytes in a dose dependent manner (data not shown). Alternatively, the insulin sensitizing effect may also be secondary to the recovery of dyslipidemia. Although bezafibrate also induced GLUT4 and CPT1b expression in skeletal muscle, IRS and PDK4 expression was not similarly effected; this together with the stable adiponectin gene expression and unrectified dyslipidemia explained why insulin resistance was not improved by bezafibrate. Additionally, differential regulation on SIRT1 gene expression constitutes another important difference between bezafibrate and ZBH. Because increased SIRT1 expression or activation could improve insulin resistance and enhance insulin secretion through deacetylation and activation of PGC-1a [35], [36], [38], [39]. Moreover, SIRT1 also could inhibit gluconeogenic gene expression through deacetylating CREB-regulated transcriptional coactivator 2 and leading to its degradation [40].

However, we unexpectedly found that 50 mg kg**^−^** ^1^ bezafibrate had little effect on hyperlipidemia in the same hamster model. Although the expression of few PPARα target genes in the liver and muscle were altered. The impact of bezafibrate on hyperlipidemic hamsters has not yet been evaluated, and a previous dose–response study with fenofibrate suggested that the dose commonly used in mouse was efficacious in hamsters [15], [17], [18], [41]. We thus reasoned this may also be the case for bezafibrate, and selected daily dosing of 25 and 50 mg kg**^−^** ^1^ bezafibrate based on earlier rodent models’ studies in which 10 mg kg**^−^** ^1^ was the lowest effective dose for lowering serum lipids in rats [29], [42]–[45]. According to present results, we thus can presume that the dyslipidemia in hamsters may be more severe than that in mice or rats. This also may partially explain why the doses of bezafibrate employed here did not improve HFD induced hyperlipidemia in the hamster in present study.

Taken together, the combination of the key pharmacophore of fibrates into the scaffold of resveratrol, ZBH represents a new compound with enhanced PPAR activation and anti-hyperlipidemic bioactivity. ZBH was found significantly ameliorate dyslipidemia and improve insulin resistance in the HFD induced hyperlipidemic hamster. These properties are a result of potent PPARα agonism, and SIRT1 promotion and activation (Figure S3). ZBH thus presents a potential new therapeutic tool to combat hyperlipidemia.

## Supporting Information

Figure S1 **Effects of ZBH on serum aspartate aminotransferase (GOT), alanine aminotransferase (GPT), creatine phosphokinase (CK) and CREA levels.** Values are mean±SE; n = 8 for NC, n = 11–12 for other groups. *P<0.05, **P<0.01, vs. MC group. NC, normal control; MC, model control; B50, Bezafibrate 50 mg kg**^−^** ^1^; Z6.25, ZBH 6.25 mg kg**^−^** ^1^; Z12.5, ZBH 12.5 mg kg**^−^** ^1^; Z25, ZBH 25 mg kg**^−^** ^1^.
(TIF)

Figure S2 **Activation of hPPARs by resveratrol.** As detailed in the “method”, activation of PPARα, δ, and γ was evaluated by transfection assays using HEK-293 cells with GAL4-hPPAR α, γ, or δ, and pUAS (5x)-tk-luc receptor vector. Results were normalized against the Renilla luciferase reading. Resveratrol shows weak and dose-dependent activation to PPAR three subtypes between 1 µM and 10 µM.
(TIF)

Figure S3 **Schematic of the physiological pathways that ameliorate dyslipidemia and insulin intolerance after ZBH treatment.** The solid black line represents direct regulation and the dotted line represents indirect effect.
(TIF)
