# Supplementary figures and images for: Novel PPAR Pan Agonist, ZBH Ameliorates Hyperlipidemia and Insulin Resistance in High Fat Diet Induced Hyperlipidemic Hamster
Source: PLoS One. 2014 Apr 23;9(4):e96056. doi: 10.1371/journal.pone.0096056 (PMC3997506; doi:10.1371/journal.pone.0096056)

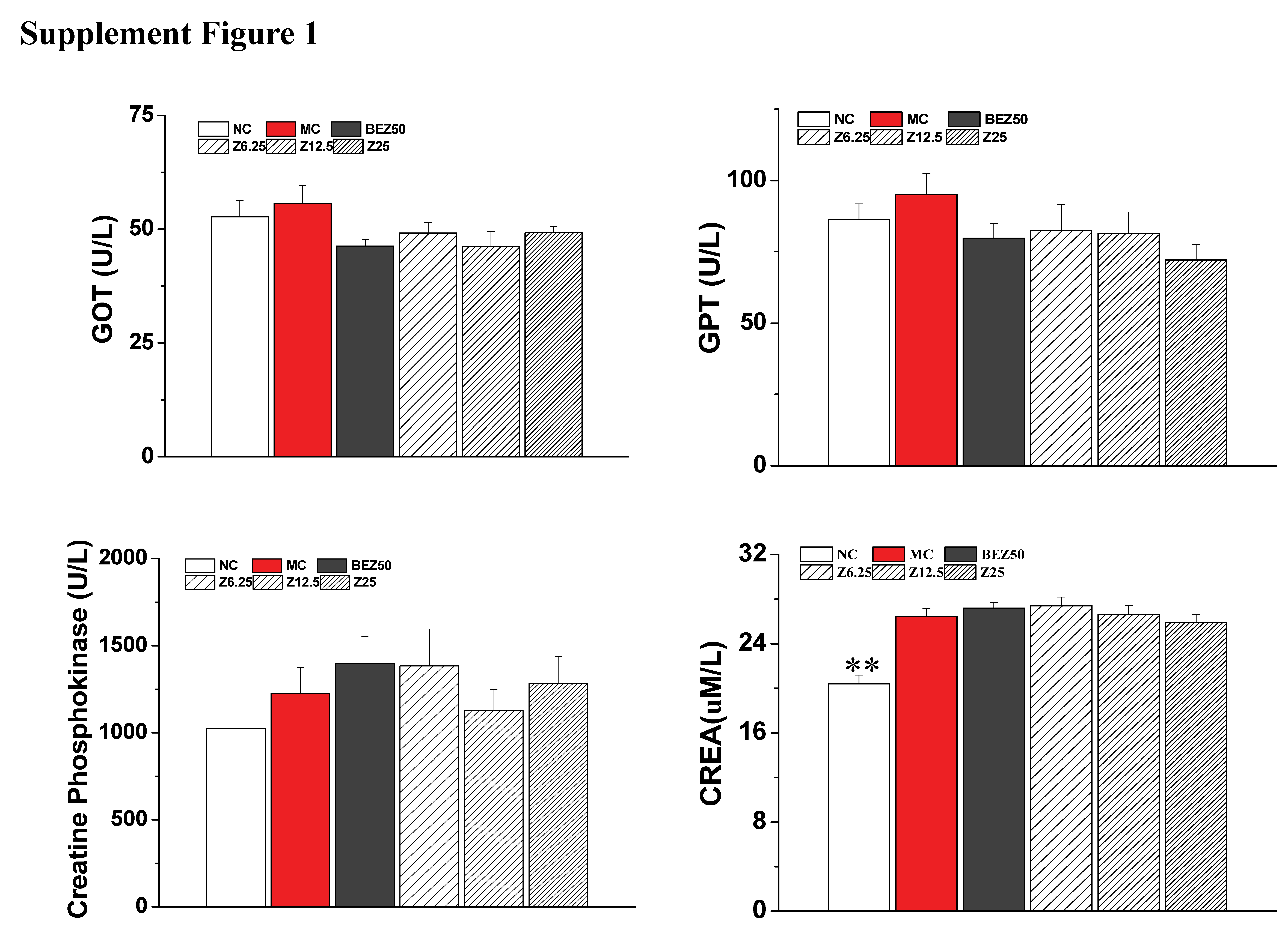

Supplement: Figure S1 — Effects of ZBH on serum aspartate aminotransferase (GOT), alanine aminotransferase (GPT), creatine phosphokinase (CK) and CREA levels. Values are mean±SE; n = 8 for NC, n = 11–12 for other groups. *P<0.05, **P<0.01, vs. MC group. NC, normal control; MC, model control; B50, Bezafibrate 50 mg kg− 1; Z6.25, ZBH 6.25 mg kg− 1; Z12.5, ZBH 12.5 mg kg− 1; Z25, ZBH 25 mg kg− 1. (TIF) [file pone.0096056.s001.tif]

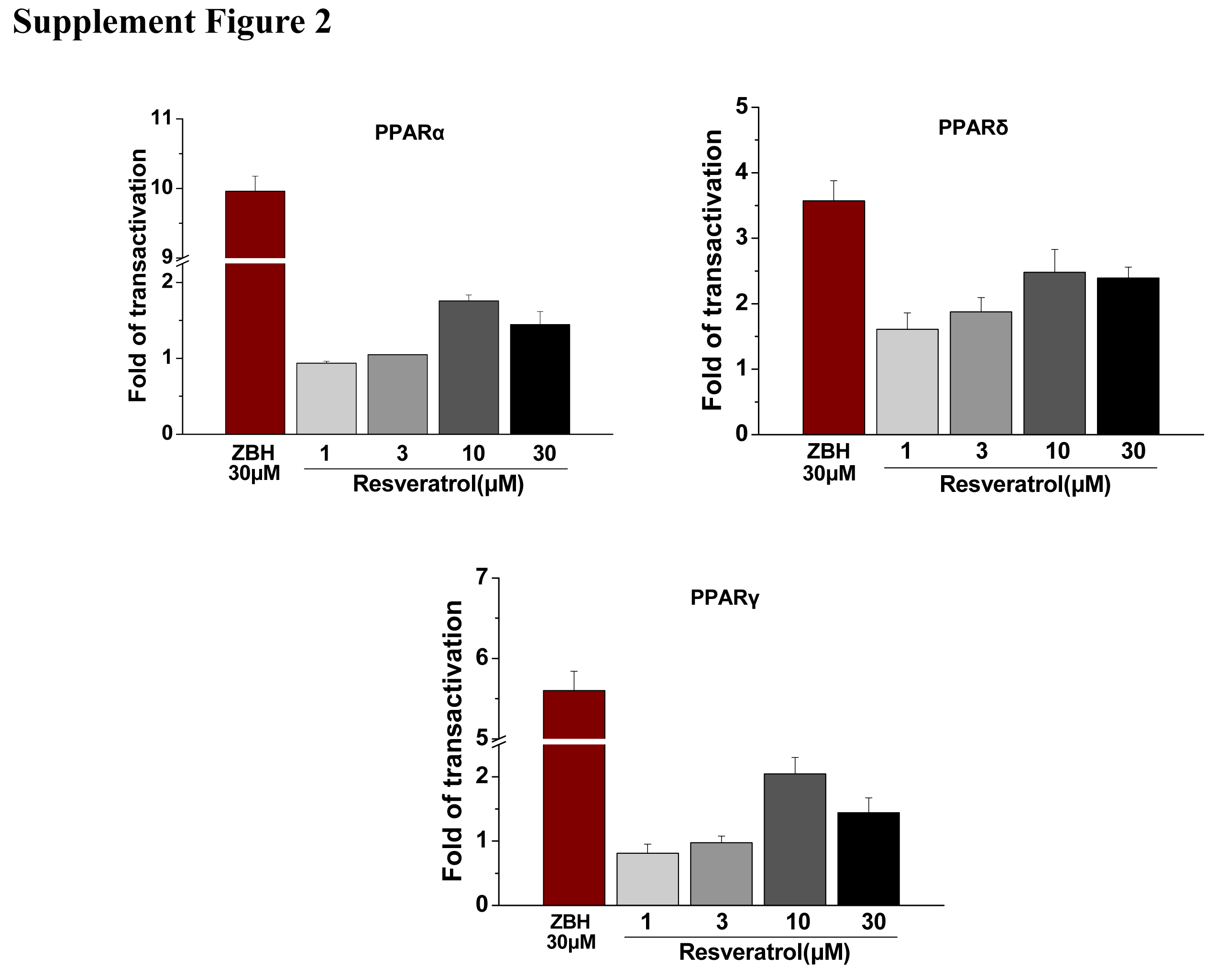

Supplement: Figure S2 — Activation of hPPARs by resveratrol. As detailed in the “method”, activation of PPARα, δ, and γ was evaluated by transfection assays using HEK-293 cells with GAL4-hPPAR α, γ, or δ, and pUAS (5x)-tk-luc receptor vector. Results were normalized against the Renilla luciferase reading. Resveratrol shows weak and dose-dependent activation to PPAR three subtypes between 1 µM and 10 µM. (TIF) [file pone.0096056.s002.tif]

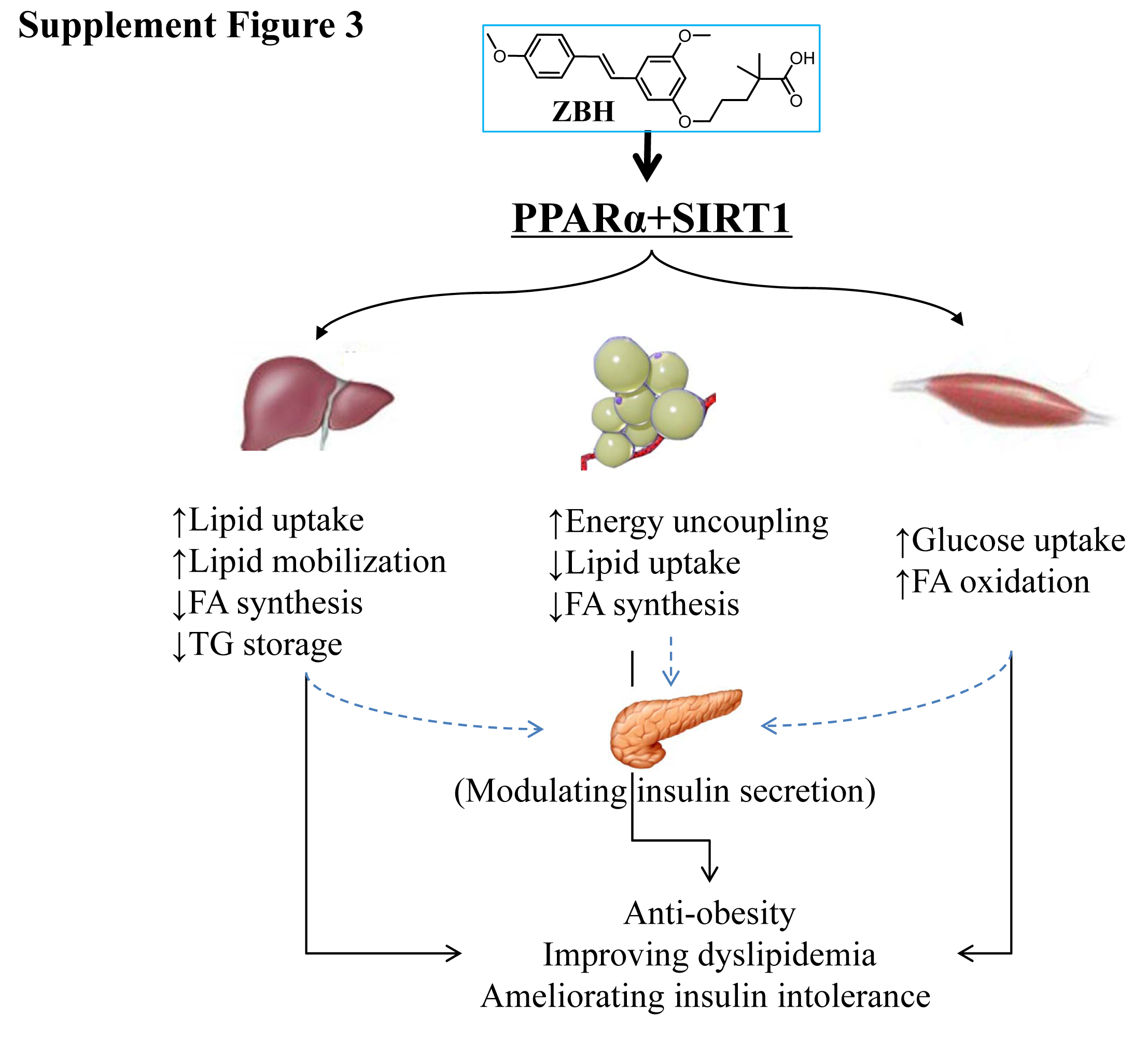

Supplement: Figure S3 — Schematic of the physiological pathways that ameliorate dyslipidemia and insulin intolerance after ZBH treatment. The solid black line represents direct regulation and the dotted line represents indirect effect. (TIF) [file pone.0096056.s003.tif]
